# Perceptions and Use of Telehealth Among Mental Health, Primary, and Specialty Care Clinicians During the COVID-19 Pandemic

**DOI:** 10.1001/jamanetworkopen.2022.16401

**Published:** 2022-06-07

**Authors:** Samantha L. Connolly, Christopher J. Miller, Allen L. Gifford, Michael E. Charness

**Affiliations:** 1 Center for Healthcare Organization and Implementation Research, VA Boston Healthcare System, Boston, Massachusetts; 2 Department of Psychiatry, Harvard Medical School, Boston, Massachusetts; 3 Boston University School of Medicine, Boston, Massachusetts; 4 Boston University School of Public Health, Boston, Massachusetts; 5 VA Boston Healthcare System, Boston, Massachusetts; 6 Harvard Medical School, Boston, Massachusetts

## Abstract

**Importance:**

Clinician attitudes toward telehealth may impact utilization rates, and findings may differ based on specialty.

**Objective:**

To determine whether clinician beliefs regarding telehealth quality and ease of use were associated with the proportion of care delivered via video, phone, and in-person across specialties.

**Design, Setting, and Participants:**

This survey study used a voluntary, anonymous survey conducted from August to September 2021 in the Department of Veterans Affairs New England Healthcare System (VANEHS). Mental health (MH), primary care (PC), and specialty care (SC) clinicians were invited to participate. Data were analyzed from October 2021 to January 2022.

**Exposures:**

Participation in a 32-item survey.

**Main Outcomes and Measures:**

The main outcomes were clinicians’ views on relative quality of video, phone, and in-person care; factors contributing to clinicians’ modality choice; telehealth challenges; and clinician modality preferences and utilization when treating new and established patients.

**Results:**

There were 866 survey respondents (estimated 64% response rate); 52 respondents reported no video or phone telehealth use in the 3 months prior to survey completion and were excluded, resulting in a final sample of 814 respondents. Respondents were divided among MH (403 respondents [49.5%]), PC (153 respondents [18.8%]), and SC (258 respondents [31.7%]). Compared with PC and SC clinicians, MH clinicians rated the quality of video care the highest (eg, compared with in-person care with masks when treating new patients: χ^2^ = 147.8; *P* < .001) and were more likely to prefer video over phone when treating both new (χ^2^ = 26.6; *P* < .001) and established (χ^2^ = 100.4; *P* < .001) patients remotely. PC and SC clinicians were more likely to rate phone care as being at least equivalent in quality to video for both new (χ^2^ = 26.3; *P* < .001) and established (χ^2^ = 33.5; *P* < .001) patients. PC and SC clinicians were also more likely to endorse challenges of video care, including patient barriers and the inability to conduct a physical examination (χ^2^ = 292.0; *P* < .001). Most PC and SC clinicians either had no preference (46 PC respondents [36.2%]; 59 SC respondents [28.4%]) or preferred phone (36 PC respondents [28.3%]; 67 SC respondents [32.2%]) for remote care of established patients. Findings aligned with utilization rates within VANEHS, with MH clinicians conducting significantly more of their encounters via video (36 734 encounters [40.3%]) than PC (3201 encounters [3.9%]) and SC (1157 encounters [4.9%]) clinicians.

**Conclusions and Relevance:**

These findings suggest that clinician attitudes regarding telehealth quality and ease of use were associated with utilization rates. Moving forward, clinician use of telehealth may be impacted by additional data regarding the relative effectiveness of modalities as well as improvements in video telehealth workflows.

## Key Points

**Question:**

Are clinician perceptions of telehealth quality associated with use?

**Findings:**

In this survey study of 866 mental health (MH), primary care (PC), and specialty care (SC) clinicians, MH clinicians rated the quality of video care the highest and were more likely to prefer video over phone when providing care for patients remotely; PC and SC clinicians were more likely to endorse challenges of video care. Findings aligned with utilization rates, with MH clinicians conducting significantly more video visits than PC and SC clinicians.

**Meaning:**

These findings suggest that specialty-specific differences in clinician perceptions of telehealth were associated with actual use.

## Teaser

This survey study assesses clinician beliefs regarding telehealth and use of telehealth modalities across specialties.

## Introduction

Rates of video and phone telehealth use skyrocketed during the COVID-19 pandemic to protect patients and clinicians from infection.^1,2^ While telehealth was most commonly used for mental health (MH) treatment prior to the pandemic, adoption increased dramatically within primary care (PC), specialty care (SC), and MH during COVID-19.^3,4^ This rapid transformation has provided an unprecedented opportunity to examine differences in telehealth utilization across specialties.

Video visits are more difficult to conduct than audio-only phone visits because they require that patients and clinicians own a video-enabled device, have internet connectivity, and know how to navigate a video telehealth platform.^5^ However, emerging evidence suggests that compared with phone visits, video visits may be more clinically effective^6,7^ and preferred by patients.^8,9^ Clinician-related factors may have greater impacts on the relative rates of video and phone use than patient factors^10^; indeed, clinicians have often been referred to as the *gatekeepers of telehealth*.^11,12^

Clinician perceptions of the quality and ease of use of virtual care modalities may contribute to variation in utilization.^13^ In a survey conducted early in the pandemic within the Veterans Health Administration (VHA), we found significant specialty-level variability in clinician perceptions, including that PC and SC clinicians may be more likely to prefer phone over video care, while MH clinicians were more likely to prefer video. We also found that clinicians expressed greater comfort in using video and phone telehealth to treat established patients compared with new patients.^14^ The survey was conducted shortly after the sudden shift to virtual care, during a time of rapid change for clinicians and health care systems.

More than 1 year later, we administered a survey with 4 objectives, aiming to understand (1) how VHA clinicians evaluate the quality of telehealth care they had delivered; (2) the factors that contribute to their choice of modality; (3) the challenges of telehealth care; and (4) their preferences for care delivery when treating both new and established patients. We also examined how utilization of in-person, phone, and video care varied by specialty and compared this variation with differences in clinician perceptions. Informed by our prior survey findings, we hypothesized that MH clinicians would have more positive perceptions of video care and would have higher rates of video use than PC and SC clinicians. This work, conducted within the largest integrated health care system in the United States, seeks to identify key clinician-level factors that may be associated with utilization of telehealth care across specialties.

## Methods

This survey study was reviewed by the VA Boston Research and Development Committee; the project was classified as quality improvement and was therefore exempt from institutional review board review. Given this status, the need for informed consent from participants was waived. This study followed the American Association for Public Opinion Research (AAPOR) reporting guideline for web-based surveys to the extent possible given our study design, in which the exact number of eligible clinicians reached by the survey invitation was unknown.

### Study Sample

A voluntary and anonymous electronic survey was emailed to MH, PC, and SC clinicians (ie, physicians, psychologists, social workers, nurse practitioners, pharmacists, physician assistants, and podiatrists) (eAppendix 1 in the Supplement) across the 8 medical centers of the VA New England Healthcare System (VANEHS), a 6-state regional health care system serving approximately 260 000 veterans annually. Among these, 4 medical centers are in urban or suburban locations and 4 medical centers are in rural locations (eAppendix 2 in the Supplement).

### Survey Design and Data Collection

Survey questions were informed by literature reviews of clinician attitudes toward telehealth,^15,16,17,18^ including a prior survey of VANEHS clinicians conducted in 2020.^14^ We assessed telehealth experience, perceptions of telehealth quality (using the National Academy of Medicine definition of quality^19^), factors contributing to the choice of care modality, challenges of telehealth care, and modality preference for remote care during the 3 months prior to survey completion. The final survey contained 32 multiple-choice questions and took approximately 10 minutes to complete (eAppendix 3 in the Supplement). A survey link was distributed by medical center chiefs of staff through clinical service chiefs to clinicians. The survey remained open from August 4, 2021, until September 20, 2021.

### VHA Outpatient Visit Data

Data for completed outpatient visits for the months of August and September 2021 were extracted from the VHA corporate data warehouse (VHA’s national clinical and administrative database^20^) and sorted according to specialty: MH, PC, and SC. Within each specialty, visits were sorted based on encounter type: in-person, video, or phone. Other care modalities, including electronic consultations, represented less than 2% of the total number of encounters and were not considered further. We analyzed data specific to the VANEHS, the region selected for surveying clinician perceptions. We also conducted secondary analyses examining outpatient visits nationally across VHA, to determine the extent to which VANEHS was representative of the VHA.

### Statistical Analysis

Descriptive statistics and χ^2^ tests were conducted using SPSS Statistics for Windows, version 26.0 (IBM). Since the exact number of eligible participants was unknown, we derived an estimated response rate (eAppendix 4 in the Supplement). *P* values were 2-sided, and statistical significance was set at *P* < .05. Data were analyzed from October 2021 to January 2022.

## Results

### Survey Population

There were 866 survey respondents across all specialties (estimated 64% response rate) (eAppendix 4 in the Supplement); 52 respondents reported no video or phone telehealth use in the 3 months prior to survey completion and were excluded from subsequent analyses. The final sample included 814 clinicians divided among MH (403 respondents [49.5%]), PC (153 respondents [18.8%]), and SC (258 respondents [31.7%]). Respondent occupations included physician (328 respondents [40.3%]), psychologist (197 respondents [24.2%]), social worker (107 respondents [13.1%]), nurse practitioner (107 respondents [13.1%]), physician assistant (36 respondents [4.4%]), pharmacist (34 respondents [4.2%]), and podiatrist (5 respondents [0.6%]). Table 1 shows the distribution of professions among specialties.

**Table 1.** Characteristics of Survey Respondents^a^

Characteristic	Respondents, No. (%) (N = 814)
Mental health	
Overall	403 (49.5)^b^
Psychologist	197 (48.9)
Social worker	107 (26.6)
Physician	63 (15.6)
Nurse practitioner	27 (6.7)
Pharmacist	6 (1.5)
Physician assistant	3 (0.7)
Primary care	
Overall	153 (18.8)^b^
Physician	87 (56.9)
Nurse practitioner	33 (21.6)
Pharmacist	25 (16.3)
Physician assistant	8 (5.2)
Specialty care	
Overall	258 (31.7)^b^
Physician	178 (69.0)
Nurse practitioner	47 (18.2)
Physician assistant	25 (9.7)
Podiatrist	5 (1.9)
Pharmacist	3 (1.2)

^a^ Specialty definitions are provided in eAppendix 1 in the Supplement.

^b^ Percentages are given out of total respondents.

During the 3 months prior to survey completion, 701 clinicians (86.1%) had conducted a phone appointment and 720 clinicians (88.5%) had conducted a video appointment, as measured via self-report. MH clinicians were significantly more likely to have completed a video appointment (386 MH clinicians [95.8%]; 137 PC clinicians [89.5%]; 197 SC clinicians [76.4%]; χ^2^ = 58.3; *P* < .001) and significantly less likely than PC or SC clinicians to have completed a phone appointment (316 MH clinicians [78.4%]; 149 PC clinicians [97.4%]; 236 SC clinicians [91.5%]; χ^2^ = 42.5; *P* < .001) in the past 3 months.

### Quality of Care: New vs Established Patients

#### Video or Phone Care vs In-Person Care With Masks

Clinicians were significantly more likely to rate video care and phone care as equivalent to or higher in quality than in-person care with masks when treating established patients compared with new patients (video: χ^2^ = 72.0, *P* < .001; phone: χ^2^ = 99.6; *P* < .001) (Table 2). Quality ratings of video care for new patients varied markedly across specialties, being highest for MH, intermediate for PC, and lowest for SC (χ^2^ = 147.8; *P* < .001). The perceived quality gap between video care for new and established patients was smallest for MH, intermediate for PC, and largest for SC. Compared with these video ratings, quality ratings of phone care vs in-person care for both new and established patients were lower and demonstrated less variation across specialties (Table 2).

**Table 2.** Clinician Perceptions of Quality of Video, Phone, and In-Person Care

Clinician specialty	Patient type	Clinicians endorsing item, No. (%)		
		Higher quality	Equivalent	Lower quality
**Video vs in-person visit**				
Overall	New^a^	127 (19.6)	271 (41.9)	249 (38.5)
	Established^b^	181 (27.1)	370 (55.4)	117 (17.5)
Mental health	New	106 (31.5)	169 (50.1)	62 (18.4)
	Established	140 (41.7)	167 (49.7)	29 (8.6)
Primary care	New	14 (12.0)	47 (40.2)	56 (47.9)
	Established	21 (16.0)	76 (58.0)	34 (26.0)
Specialty care	New	7 (3.6)	55 (28.5)	131 (67.9)
	Established	20 (10.0)	127 (63.2)	54 (26.9)
**Phone vs in-person visit**				
Overall	New^c^	41 (6.2)	150 (22.8)	466 (70.9)
	Established^d^	48 (7.0)	329 (48.1)	307 (44.9)
Mental health	New	27 (8.5)	74 (23.3)	217 (68.2)
	Established	28 (8.6)	144 (44.3)	153 (47.1)
Primary care	New	10 (8.1)	33 (26.8)	80 (65.0)
	Established	11 (8.2)	72 (53.7)	51 (38.1)
Specialty care	New	4 (1.9)	43 (19.9)	169 (78.2)
	Established	9 (4.0)	113 (50.2)	103 (45.8)
**Phone vs video**				
Overall	New^e^	24 (3.7)	183 (28.4)	438 (67.9)
	Established^f^	30 (4.5)	336 (50.2)	303 (45.3)
Mental health	New	11 (3.4)	65 (20.0)	249 (76.6)
	Established	9 (2.7)	135 (41.0)	185 (56.2)
Primary care	New	6 (5.0)	50 (41.3)	65 (53.7)
	Established	9 (6.9)	81 (61.8)	41 (31.3)
Specialty care	New	7 (3.5)	68 (34.2)	124 (62.3)
	Established	12 (5.7)	120 (57.4)	77 (36.8)

^a^ Difference among specialties in quality ratings: χ^2^ = 147.8; *P* < .001.

^b^ Difference among specialties in quality ratings: χ^2^ = 88.9; *P* < .001.

^c^ Difference among specialties in quality ratings: χ^2^ = 14.2; P < .01.

^d^ Difference among specialties in quality ratings: χ^2^ = 8.2.

^e^ Difference among specialties in quality ratings: χ^2^ = 26.3; *P* < .001.

^f^ Difference among specialties in quality ratings: χ^2^ = 33.5; *P* < .001.

#### Phone Care vs Video Care

Less than one-third of clinicians (207 clinicians [32.1%]) rated phone as equivalent to or higher in quality than video when treating new patients (Table 2). Endorsement increased significantly when considering established patients (χ^2^ = 68.3; *P* < .001). PC and SC clinicians were significantly more likely than MH clinicians to rate phone care as equivalent or higher quality than video care for new (χ^2^ = 26.3; *P* < .001) and established (χ^2^ = 33.5; *P* < .001) patients.

### Clinician-Reported Major Contributors to Modality Choice

Clinicians were asked to endorse the major contributors to their decision to choose video, phone, or in-person care (ie, modality choice) (Table 3). Overall, patient preference was the most frequently endorsed factor (531 respondents [73.3%]) followed by clinical judgment (408 respondents [56.7%]) and leadership guidance (214 respondents [30.7%]). There were some notable differences across specialties; PC clinicians were more likely than MH or SC clinicians to describe scheduling staff as having a major influence on modality choice (χ^2^ = 36.6; *P* < .001). MH clinicians were more likely to report that choices were impacted by leadership guidance (χ^2^ = 27.4; *P* < .001) and available data regarding the relative effectiveness of the modalities (χ^2^ = 56.4; *P* < .001). SC clinicians were more likely to endorse clinical judgment (χ^2^ = 18.1; *P* = .001) and less likely to endorse patient preference (χ^2^ = 15.4; *P* = .004) as contributors.

**Table 3.** Clinician Endorsement of Major Contributors to Modality Choice and Significant Challenges of Telehealth Use

Item	Clinicians endorsing item, No. (%)				χ^2^
	Overall	MH	PC	SC	
**Modality choice**					
Patient preference	531 (73.3)	278 (78.3)	104 (75.9)	149 (64.2)	15.4^a^
Clinical judgment	408 (56.7)	194 (55.1)	70 (51.5)	144 (62.3)	18.1^a^
Leadership guidance	214 (30.7)	129 (36.8)	34 (26.2)	51 (23.5)	27.4^b^
Available data/research comparing effectiveness of modalities	124 (18.6)	90 (26.5)	13 (10.3)	21 (10.4)	56.4^b^
Scheduler preference/messaging	88 (13.6)	34 (11.0)	29 (23.4)	25 (11.7)	36.6^b^
Workload credit	57 (8.4)	38 (11.3)	8 (6.0)	11 (5.1)	8.3
**Challenges to modality use^c^**					
Phone					
Inability to conduct a physical examination to the degree required	247 (33.9)	42 (11.6)	57 (41.9)	148 (63.8)	398.3^b^
Assessing physical health status	209 (28.7)	80 (22.3)	34 (25.0)	95 (40.9)	127.1^b^
Receiving full workload credit for appointments	148 (20.5)	57 (16.0)	22 (16.3)	69 (30.0)	32.4^b^
Video					
Patient difficulty with their device or platform	239 (33.7)	92 (26.0)	46 (34.8)	101 (45.1)	58.2^b^
Lack of technical support/training for patients	206 (29.0)	83 (23.4)	39 (29.5)	84 (37.5)	60.6^b^
Patient access to adequate internet	202 (28.5)	83 (23.4)	39 (29.5)	80 (35.9)	32.4^b^
Lack of technical support/training for patients who received a VHA tablet	135 (19.0)	69 (19.5)	19 (14.4)	47 (21.0)	43.6^b^
Inability to conduct a physical examination to the degree required	119 (16.7)	20 (5.6)	25 (18.9)	74 (32.9)	292.0^b^
Converting an ongoing visit from phone to video	100 (14.1)	32 (9.1)	26 (20.0)	42 (18.6)	56.1^b^
Scheduling processes	80 (11.3)	26 (7.4)	23 (17.4)	31 (13.7)	48.5^b^
Ordering patient a tablet if needed	73 (10.2)	28 (7.9)	10 (7.6)	35 (15.5)	50.2^b^

Abbreviations: MH, mental health; PC, primary care; SC, specialty care; VHA, Veterans Health Administration.

^a^ Significant difference by specialty via χ^2^: *P* < .01.

^b^ Significant difference by specialty via χ^2^: *P* < .001.

^c^ Item values are presented if at least 15% of 1 of the specialty groups endorsed it as a significant challenge. The full survey is presented in eAppendix in the Supplement.

### Challenges of Phone and Video Use

SC clinicians were more likely than PC or MH clinicians to endorse significant challenges of phone appointments, including the inability to assess physical health status (χ^2^ = 127.1; *P* < .001), conduct an adequate physical examination (χ^2^ = 398.3; *P* < .001), and receive full workload credit (χ^2^ = 32.4; *P* < .001). With regards to video appointments, PC and SC clinicians generally endorsed challenges at higher rates than MH clinicians (Table 3). Among these were clinician challenges, such as the inability to conduct an adequate physical examination (χ^2^ = 292.0; *P* < .001), and patient challenges, such as patient difficulty using their device or telehealth platform (χ^2^ = 58.2; *P* < .001), lack of technical support and training for patients (χ^2^ = 60.6; *P* < .001), and inadequate internet connectivity (χ^2^ = 32.4; *P* < .001) (Table 3).

### Clinician Preferences When Providing Care for Patients Remotely

Overall, when asked to select the modality they would prefer to use while delivering remote care, most clinicians expressed a preference for video over phone, particularly for new vs established (χ^2^ = 80.3; *P* < .001) patients (Table 4). MH clinicians showed the strongest preference for video over phone for both new (χ^2^ = 26.6; *P* < .001) and established (χ^2^ = 100.4; *P* < .001) patients and the smallest distinction between new and established patients. In contrast, most PC and SC clinicians either had no preference (46 PC respondents [36.2%]; 59 SC respondents [28.4%]), or preferred phone (36 PC respondents [28.3%]; 67 SC respondents [32.2%]) when treating established patients (Table 4).

**Table 4.** Clinician Modality Preference When Caring for New and Established Patients Remotely

Clinician specialty	Patient type	Clinicians endorsing, No. (%)		
		Prefer video	Prefer phone	No preference
Overall	New^a^	514 (78.6)	46 (7.0)	94 (14.4)
	Established^b^	379 (56.1)	129 (19.1)	168 (24.9)
Mental health	New	291 (86.4)	15 (4.5)	31 (9.2)
	Established	252 (73.9)	26 (7.6)	63 (18.5)
Primary care	New	81 (66.9)	12 (9.9)	28 (23.1)
	Established	45 (35.4)	36 (28.3)	46 (36.2)
Specialty care	New	142 (72.4)	19 (9.7)	35 (17.9)
	Established	82 (39.4)	67 (32.2)	59 (28.4)

^a^ Difference among specialties in quality ratings: χ^2^ = 26.6; *P* < .001.

^b^ Difference among specialties in quality ratings: χ^2^ = 100.4; *P* < .001.

### Specialty-Specific Use of Telehealth Modalities

The survey provided specialty-specific assessments of quality and preferences for the use of in-person, video, and phone care. We next determined the actual use of these care modalities over the 2 months when the survey was conducted. During this time, VANEHS recorded 402 989 completed visits (91 314 MH visits [22.7%]; 82 946 PC visits [20.6%]; 228 729 SC visits [56.8%]), of which 358 470 visits (89.0%) were for established patients, including 89 236 MH visits (97.7%), 79 113 PC visits (95.4%), and 190 121 SC visits (83.1%).

Across specialties, new patients received care in-person at higher rates than established patients (χ^2^ = 4494.8; *P* < .001). VANEHS data also revealed significant, specialty-specific differences in the proportions of video, phone, and in-person encounters (Table 5). MH provided the highest percentage of video visits for both new (χ^2^ = 3987.4; *P* < .001) and established (χ^2^ = 81 345.0; *P* < .001) patients, with video encounters accounting for 36 734 (40.3%) of all MH encounters, compared with 3201 PC encounters (3.9%) and 11 157 SC encounters (4.9%). When considering established patients, SC provided the most in-person care, while PC demonstrated the highest rates of phone care across specialties (χ^2^ = 81345.0; *P* < .001). Utilization rates and specialty-level differences observed within VANEHS were very similar to those seen nationally across VHA (eTable in the Supplement).

**Table 5.** VA New England Healthcare System Completed Visits During August and September 2021 by Modality

Specialty	Patient type	Overall completed visits, No. (%)^a^		
		In-person	Phone	Video
Overall	New^b^	37 975 (85.3)	2933 (6.6)	2386 (5.4)
	Established^c^	259 735 (72.5)	47 874 (13.4)	48 706 (13.6)
Mental health	New	1170 (56.3)	150 (7.2)	745 (35.9)
	Established	40 902 (45.8)	11 829 (13.3)	35 989 (40.3)
Primary care	New	3259 (85.0)	318 (8.3)	249 (6.5)
	Established	58 384 (73.8)	17 423 (22.0)	2952 (3.7)
Specialty care	New	33 546 (86.9)	2465 (6.4)	1392 (3.6)
	Established	160 449 (84.4)	18 622 (9.8)	9765 (5.1)

^a^ Electronic consultations comprised a small proportion of completed visits and are not shown; therefore, some categories do not sum to 100%.

^b^ Difference among specialties in percentage of encounters completed in person, by phone, or by video: χ^2^ = 3987.4; *P* < .001.

^c^ Difference among specialties in percentage of encounters completed in person, by phone, or by video: χ^2^ = 81 345.0; *P* < .001.

## Discussion

This survey study of VHA clinicians found substantial specialty-level differences in clinician beliefs regarding the quality of video and phone telehealth, major contributors to their modality choice, challenges of telehealth use, and modality preferences when providing care remotely. These findings may in part explain observed differences in actual video, phone, and in-person care utilization across specialties.

MH clinicians, who provided the greatest proportion of video visits at the time of the survey, rated the quality of video care the highest and were more likely to prefer video over phone when providing care for patients remotely. These findings align with prior work reporting high MH clinician satisfaction with video telehealth, particularly as they gain experience with the modality.^15,21^ MH clinicians were also more likely to report that their selection of care modalities was influenced by leadership guidance and data regarding the relative effectiveness of video, phone, and in-person care. Indeed, given that telehealth was being used for MH care well before the onset of the COVID-19 pandemic, there is a strong body of evidence demonstrating that video care is noninferior to in-person MH services,^22,23,24^ as well as an emerging literature suggesting that phone care may sometimes be inferior in quality to video care.^7,25,26^ Findings from this survey suggest that clinician and perhaps leadership decision-making has been influenced by these data. There is less literature regarding telehealth effectiveness in PC and SC, but publication of high-quality studies has increased since the start of the pandemic^27^; this work will be critical in informing PC and SC clinicians’ and leadership’s decision-making regarding the choice of care modalities.

PC and SC clinicians, who conducted substantially less video care than MH, had multiple similarities in their responses across the survey. These clinicians were more likely to rate phone care as being at least equivalent in quality to video. They were also more likely to endorse challenges of video care, including patient barriers to use and the inability to conduct an adequate physical examination. Importantly, most PC and SC clinicians either had no preference or preferred phone for remote care of established patients. However, there were some notable differences between PC and SC clinicians. SC clinicians were more likely to endorse their clinical judgment as influencing modality choice. They also were more likely to rate video and phone care as being lower in quality than in-person care when treating new patients. In addition, SC clinicians endorsed more challenges with phone visits compared with PC clinicians, including an inability to assess health status.

These findings may partly explain why SC clinicians conducted the highest proportion of in-person visits across all clinician groups. SC clinicians may be more likely to view both video and phone care as inferior to in-person care because of the limited ability to conduct physical examinations and assess patient health status. Indeed, other clinician surveys have identified difficulties in conducting physical examinations and performing procedures as barriers to remote SC.^28,29,30^

PC clinicians conducted the highest proportion of phone visits when providing care for established patients. This could be owing, in part, to their increased likelihood of endorsing challenges of video care coupled with a tendency to believe that video and phone care are equivalent in quality, particularly for established patients. Indeed, most PC clinicians either preferred phone or had no preference between phone and video for the remote care of established patients. This finding underscores the importance of complexity in influencing adoption of new technologies^13^; if PC clinicians believe that phone and video care are equivalent in quality, ease of use may then drive the choice of phone over video, particularly when treating patients whom they have already seen in-person.

Most clinicians across specialties endorsed patient preference as a major contributor to modality choice. Yet while patient preference for video over phone visits becomes increasingly apparent,^8,9^ utilization data reveal a large portion of remote visits continue to be conducted by phone. The extent to which patient preference is in fact a post hoc rationalization for clinician preference is unknown. Importantly, research conducted during the pandemic has underlined the substantial role of clinicians in influencing rates of video use. One study found that one-third of Medicare enrollees were only offered phone visits, and not video, for remote appointments, despite the fact that most of them owned a video-enabled device.^31^ Another study demonstrated that practice- and clinician-level factors explained significantly more of the variation in video visit utilization than did patient-level factors.^10^ The findings of these studies^10,31^ highlight the need to more closely examine the extent to which patient preference is being fully incorporated into the decision-making process when choosing a care modality.

Likewise, it is unclear how often what we refer to as patient preference is instead a measure of patient readiness for telehealth (ie, that the patient owns a video-enabled device or is comfortable navigating a telehealth platform). A patient without a smartphone may be viewed as preferring a phone appointment because they do not have access to the appropriate technologies. Indeed, COVID-19 has revealed a stark digital divide in which patients who are older and/or have lower income are less likely to be video-ready.^32,33,34,35^ These findings highlight the importance of increasing patient access to video-enabled devices and broadband connectivity to ensure that they are able to successfully engage in video visits. The VHA’s tablet distribution program^36^ and the Federal Communications Commissions’ Lifeline program,^37^ which offers discounted broadband to individuals with low income, are important steps in this direction. Increasing technical support staff to help patients troubleshoot technology will be critical, particularly for clinicians with large caseloads and short appointment times. Broadly, processes and workflows must be streamlined to ensure that video visits are as simple and accessible as possible for both patients and clinicians.

### Limitations

This study has some limitations, including its use of a regional sample of VHA clinicians. Whereas utilization patterns within VANEHS closely mirrored national VHA data, it is possible that attitudes may differ across regions. Given that this survey study was conducted within a national integrated health care system, results may not fully generalize to alternative settings. However, it is important to note that VHA’s financial model is also a strength of this study. Because clinician choices in VHA are not driven by fee-for-service reimbursement schedules, they may more closely reflect intrinsic clinician preferences.^38,39,40^ In addition, we did not collect demographic information from respondents, including sex, age, or race or ethnicity, in an effort to keep the length of the survey manageable and to ensure anonymity. However, this information could provide additional insights and is worth examining in future work.

## Conclusions

This survey study found significant specialty-level differences in clinician attitudes toward video and phone telehealth care, many of which aligned with observed differences in actual utilization of these modalities. Our findings suggest that in the absence of financial incentives, clinician beliefs, particularly regarding the quality and ease of use of telehealth, played an important role in the care modalities that were ultimately used with patients. There is a need for additional data regarding the relative effectiveness of video and phone telehealth as well as improved processes to better integrate video telehealth into clinician workflows. Such advances will be critical in influencing clinician attitudes and ensuring the provision of high-quality care at the right place and the right time.

## Notes

Supplement. **eAppendix 1.** Sample Definitions **eAppendix 2.** VA New England Healthcare System Medical Centers **eAppendix 3.** Full Survey **eAppendix 4.** Estimated Response Rate Calculation **eTable.** National VHA Completed Visits During August and September 2021
