# Supplementary material for: Perceptions and Use of Telehealth Among Mental Health, Primary, and Specialty Care Clinicians During the COVID-19 Pandemic
Source: JAMA Netw Open. 2022 Jun 7;5(6):e2216401. doi: 10.1001/jamanetworkopen.2022.16401 (PMC9175071; doi:10.1001/jamanetworkopen.2022.16401)
Supplement: Supplement. — eAppendix 1. Sample Definitions eAppendix 2. VA New England Healthcare System Medical Centers eAppendix 3. Full Survey eAppendix 4. Estimated Response Rate Calculation eTable. National VHA Completed Visits During August and September 2021 [file jamanetwopen-e2216401-s001.pdf]

Supplemental Online Content

Connolly SL, Miller CJ, Gifford AL, Charness ME. Perceptions and use of telehealth among mental health, primary, and specialty care clinicians during the COVID-19 pandemic. *JAMA Netw Open*. 2022;5(6):e2216401. doi:10.1001/jamanetworkopen.2022.16401

eAppendix 1. Sample Definitions

eAppendix 2. VA New England Healthcare System Medical Centers

eAppendix 3. Full Survey

eAppendix 4. Estimated Response Rate Calculation

eTable. National VHA Completed Visits During August and September 2021

This supplemental material has been provided by the authors to give readers additional information about their work.

© 2022 Connolly SL et al. *JAMA Network Open.*

eAppendix 1. Sample Definitions

Mental Health (MH)
Providers: Physicians, psychologists, social workers, PharmDs, nurse practitioners, and physician assistants

Primary Care (PC)
Primary Care, Women's Health, and General Internal Medicine

Providers: Physicians, PharmDs, nurse practitioners, and physician assistants

Specialty Care (SC)
Anesthesiology, Cardiology, Cardiothoracic Surgery, Dermatology, Endocrinology, ENT, Gastroenterology, General Surgery, Geriatrics, Gynecology, Hematology/Oncology, Infectious Disease, Nephrology, Neurology, Neurosurgery, Ophthalmology, Orthopedics, Pain Medicine, Palliative care/Hospice, Plastic Surgery, Podiatry, Pulmonary/Allergy/Sleep/Critical Care Medicine, Radiology, Rehabilitation Medicine, Rheumatology, Spinal Cord Injury, Urgent Care/Emergency Medicine, Urology, Vascular surgery

Providers: Physicians, PharmDs, nurse practitioners, physician assistants, and podiatrists

© 2022 Connolly SL et al. *JAMA Network Open.*

eAppendix 2. VA New England Healthcare System Medical Centers

Urban/suburban sites:
VA Bedford Healthcare System; Bedford, MA
Providence VA; Providence, RI
VA Boston Healthcare System; West Roxbury, MA
VA Connecticut Healthcare System; West Haven, CT

Rural sites:
Manchester VA Medical Center; Manchester, NH
VA Central Western Massachusetts Healthcare System; Northampton, MA
VA Maine Healthcare System; Augusta, ME
White River Junction VA; White River Junction, VT

© 2022 Connolly SL et al. *JAMA Network Open.*

eAppendix 3. Full Survey

1. Select the location you are affiliated with.

    a. Bedford
    b. Boston
    c. Central Western Massachusetts
    d. Connecticut
    e. Maine
    f. Manchester
    g. Providence
    h. White River Junction

2. Select your primary work setting. (For example, if your clinic is located in a CBOC, choose CBOC, even if you are currently working from home)
    a. Main hospital/multi-campus location
    b. CBOC
    c. Other (please specify)

3. Are you a full-time or part-time VA employee?
    a. Full-time
    b. Part-time
    c. Other (please specify)

4. Please indicate your occupation.
    a. Audiologist
    b. CNS
    c. Dentist
    d. LPN
    e. Nurse practitioner
    f. Occupational therapist
    g. Optometrist
    h. PA
    i. PharmD
    j. Physical therapist
    k. Physician
    l. Podiatrist
    m. Psychologist
    n. RN
    o. Social worker
    p. Speech pathologist
    q. Other (please specify)

© 2022 Connolly SL et al. *JAMA Network Open.*

5. What is the main type of outpatient service you provide? (please select only one option)
   a. Anesthesiology
   b. Audiology
   c. Cardiology
   d. Cardiothoracic surgery
   e. Dental
   f. Dermatology
   g. Endocrinology
   h. ENT
   i. Gastroenterology
   j. General Internal Medicine
   k. General Surgery
   l. Geriatrics
   m. Gynecology
   n. Hematology/Oncology
   o. Home Based Primary Care
   p. Homeless Outreach/Housing
   q. Infectious Disease
   r. Mental Health
   s. Nephrology
   t. Neurology
   u. Neurosurgery
   v. Occupational therapy
   w. Ophthalmology
   x. Optometry
   y. Orthopedics
   z. Pain Medicine
   aa. Palliative Care/Hospice
   bb. Physical Therapy
   cc. Plastic Surgery
   dd. Podiatry
   ee. Primary Care
   ff. Prosthetics
   gg. Pulmonary, Allergy, Sleep, Critical Care Medicine
   hh. Radiology
   ii. Rehabilitation Medicine
   jj. Rheumatology
   kk. Speech Pathology/Speech Therapy
   ll. Spinal Cord Injury

© 2022 Connolly SL et al. *JAMA Network Open.*

mm. Urology
nn. Vascular Surgery
oo. Women's Health
pp. Other (please specify)

6. Please estimate the percentage of your total outpatient appointments that you completed in-person in the past 3 months.
   0-100% in 10% increments

7. Please estimate the percentage of your total outpatient appointments that you completed by phone in the past 3 months.
   0-100% in 10% increments

8. Please estimate the percentage of your total outpatient appointments that you completed by video telehealth in the past 3 months. (e.g., VA Video Connect, Doximity Video, Zoom, Facetime, WebEx, or similar)
   0-100% in 10% increments

The next questions refer to **quality of care**. Quality refers to whether care is effective, safe, timely, efficient, equitable, and patient-centered.

9. Rate the quality of care you deliver via **video telehealth** to **new** patients.
   a. lower quality than in-person with masks/ equivalent to in-person with masks/ higher than in-person with masks/ N/A

10. Rate the quality of care you deliver via **video telehealth** to **established** patients.
   a. lower quality than in-person with masks/ equivalent to in-person with masks/ higher than in-person with masks/ N/A

11. Rate the quality of care you deliver via **phone** to **new** patients.
   a. lower quality than in-person with masks/ equivalent to in-person with masks/ higher than in-person with masks/ N/A
   b. lower quality than video / equivalent to video/ higher than video/ N/A

12. Rate the quality of care you deliver via **phone** to **established** patients.
   a. lower than in-person with masks/ equivalent to in-person with masks/ higher than in-person with masks/ N/A
   b. lower quality than video / equivalent to video/ higher than video/ N/A
13. Please rate how each of the following factors contribute to which care modality (phone, video, or in-person) you use with a given patient.

© 2022 Connolly SL et al. *JAMA Network Open.*

Major contributor Somewhat of a contributor Not a contributor

- My clinical judgment
- Patient preference
- Scheduler preference/messaging
- Leadership guidance
- Workload credit
- Available data/research comparing effectiveness of the modalities
- Other (Please specify)

14. Please rate the following aspects of conducting **phone** appointments in the past 3 months.

Not a challenge Somewhat of a challenge Significant challenge N/A

- Hearing the patient adequately
- Patient hearing me adequately
- Establishing rapport with the patient
- Ensuring patient's safety and confidentiality
- Assessing physical health status
- Inability to conduct a physical exam to the degree required
- Receiving full workload credit for appointments
- Poor communication/support from leadership re: phone appointments
- Other

15. What percentage of the time have you experienced a technology issue when conducting a **phone** appointment in the past 3 months? (e.g., poor audio, call dropped)

    0-100% in 10% increments

16. Please rate your satisfaction with the following aspects of VA Video Connect in the past 3 months.

Very dissatisfied Dissatisfied Neutral Satisfied Very Satisfied N/A

- Ease of use
- Coordination/scheduling of appointments
- Audio and video quality
- Overall satisfaction

17. What percentage of the time have you experienced a technology issue when conducting a VA Video Connect appointment in the past 3 months? (e.g., poor audio or video, call dropped, patient or provider unable to see or hear each other, link does not work)

© 2022 Connolly SL et al. *JAMA Network Open.*

0-100%, increments of 10%

18. What percentage of your video telehealth appointments have been successfully completed via VA Video Connect in the past 3 months? (e.g., did not have to convert to a phone visit or to an alternate video telehealth platform, or reschedule due to technological difficulties)
    0-100%, 10% increments

19. Have you used any other platforms to conduct video telehealth appointments with your VA patients in the past 3 months?
    I have not used any other telehealth platforms
    Doximity Video
    Facetime
    Cisco Webex Meetings/ Webex Teams
    Microsoft Teams
    Skype (standard)
    Skype for Business
    Zoom (standard)
    Zoom for Healthcare
    GoToMeeting
    Doxy.me
    Google Duo
    Other (please specify)

20. Do you/would you prefer using any of the following platforms over VA Video Connect? Select all that apply:
    No, I do not prefer an alternative platform.
    Doximity Video
    Facetime
    Cisco Webex Meetings/ Webex Teams
    Microsoft Teams
    Skype (standard)
    Skype for Business
    Zoom (standard)
    Zoom for Healthcare
    GoToMeeting
    Doxy.me
    Google Duo
    Other (please specify)
21. Please select reasons why you prefer an alternative video telehealth platform(s) over VA Video Connect (select all that apply):
    Easier to use

© 2022 Connolly SL et al. *JAMA Network Open.*

Better audio/video quality
More features (e.g., virtual backgrounds, better grid views of multiple attendees, allows for call-in by phone only)
More reliable (e.g., Calls do not drop, or do not drop as often)
I'm already familiar with the platform
VA Video Connect does not work on my device
VA Video Connect does not work on patient's device
Patients already familiar with the platform
Patients requested to use the platform
Other (please specify)
N/A

22. Please rate the following technical aspects on the **provider side** when conducting video telehealth appointments in the past 3 months.

Not a challenge Somewhat of a challenge Significant challenge N/A

Hearing the patient adequately
Seeing the patient adequately
Having access to adequate internet connection (provider side)
Difficulty using my device and/or using video telehealth platforms
Scheduling processes
Converting an ongoing visit from phone to video
Receiving full workload credit for appointments
Placing a "digital divide consult" to order the patient a tablet if needed
Lack of technical support/training for providers
Insufficient communication/support from leadership re: telehealth

23. Please rate the following technical aspects on the **patient side** when conducting video telehealth appointments in the past 3 months.

Not a challenge Somewhat of a challenge Significant challenge N/A

Patient hearing me adequately
Patient seeing me adequately
Patient having access to adequate internet connection
Patient having difficulty using their device and/or using video telehealth platforms
Lack of general technical support/training for patients (e.g. test calls before appointment)
Lack of technical support/training for patients **who have received a tablet via the digital divide consult** (e.g. assistance with tablet set up)

© 2022 Connolly SL et al. *JAMA Network Open.*

24. Please rate these additional aspects of conducting video telehealth appointments in the past 3 months.

    Not a challenge Somewhat of a challenge Significant challenge N/A

    Establishing rapport with the patient
    Ensuring patient's safety and confidentiality
    Assessing physical health status
    Inability to conduct a physical exam to the degree required
    Inappropriate or distracting patient behavior (e.g. patient not properly clothed, in bed, children/pets interrupting appointment)

25. When caring for **new** patients remotely, do you generally prefer conducting phone visits or video telehealth visits?

    Phone visits / Video telehealth visits/No preference/ Other (please specify)

26. When caring for **established** patients remotely, do you generally prefer conducting phone visits or video telehealth visits?

    Phone visits / Video telehealth visits/No preference/ Other (please specify)

27. In your estimation, what percentage of your patients prefer/request **phone care** (over video or in-person)?

    0-100%, 10% increments Don't know N/A

28. In your estimation, what percentage of your patients prefer/request **video care** (over phone or in-person)?

    0-100%, 10% increments Don't know N/A

29. Moving forward, what percentage of **new** patients would you be comfortable caring for using the following modalities:

    0-100%, 10% increments N/A

    a. In-person (patient/provider wearing masks)
    b. Phone
    c. Video telehealth

30. Moving forward, what percentage of **established** patients would you be comfortable caring for using the following modalities:

    0-100%, 10% increments N/A

    a. In-person (patient/provider wearing masks)

© 2022 Connolly SL et al. *JAMA Network Open.*

b. Phone
c. Video telehealth

31. Please rate your satisfaction conducting video telehealth and/or phone appointments **from your own home** in the past 3 months.

    Very dissatisfied Dissatisfied Neutral Satisfied Very satisfied N/A

32. What percentage of the time would you prefer to provide remote care (via phone or video) from **your own home or other personal location?**

    0% (I prefer providing all remote care from my office) -100%, 10% increments N/A

If provider answers anything other than 0% on question 32:

Please rate how each of the following factors contribute to your preference for providing remote care from your own home or other personal location.

Major contributor Somewhat of a contributor Not a contributor

- Saves time not having to commute to work
- Saves money not having to commute to work
- Lack of available/suitable office space at the VA
- Lack of parking at the VA
- More comfortable at home/other personal location (e.g., access to comfortable furniture, temperature control, kitchen)
- Responsibilities caring for family members/pets

33. Please provide any suggestions regarding how VISN 1 can make virtual care more accessible and satisfactory.

SURVEY ENDS

© 2022 Connolly SL et al. *JAMA Network Open.*

eAppendix 4. Estimated Response Rate Calculation

Given that survey links were distributed to providers via the service chiefs of their respective medical centers through separate email listservs, the exact number of eligible providers reached by the survey invitation is unknown, and we are therefore unable to calculate an exact response rate. The number of overall active providers within the included services and provider categories (see Supplement 1) in the thirty days prior to data analysis was derived from the VA Support Service Center Capital Assets (VSSC) operations database (n=1354). The estimated response rate was calculated by dividing the number of survey respondents (n=866) by this number of active providers (n=1354), yielding a value of 64%.

© 2022 Connolly SL et al. *JAMA Network Open.*

eTable. National VHA Completed Visits During August and September 2021

Specialty	Patient type	N (%) of overall completed visits		
		**In-person**	**Phone**	**Video**
Overall	New	937064 (80.9)	92063 (7.9)	80060 (6.9)
	Established	5602052 (68.3)	1279235 (15.6)	1208143 (14.7)
Mental health	New	26385 (48.4)	10805 (19.8)	16349 (30.0)
	Established	580938 (34.4)	310769 (18.4)	768164 (45.5)
Primary care	New	87822 (78.7)	13301 (11.9)	9548 (8.6)
	Established	1471210 (66.2)	594860 (26.8)	140102 (6.3)
Specialty care	New	822857 (82.9)	67957 (6.8)	54163 (5.5)
	Established	354990 4 (82.8)	373606 (8.7)	299877 (7.0)

Note: N= 9,198,617. Electronic consultations comprised a small percentage of completed visit and are not shown; therefore, some categories do not sum to 100% (see Methods section for further detail).

Across specialties, new patients received care in-person at higher rates than established patients (χ^2 = 110248.2, p < .0001). MH provided the highest percentage of video visits, for both new (χ^2 = 64829.3, p < 0.001) and established (χ^2 =2103204, p < .0001) patients. When considering established patients, SC provided the most in-person care, while PC demonstrated the highest rates of phone care across specialties (χ^2 =2103204, p < .0001).

© 2022 Connolly SL et al. *JAMA Network Open.*
